# NanoSIMS and tissue autoradiography reveal symbiont carbon fixation and organic carbon transfer to giant ciliate host

**DOI:** 10.1038/s41396-018-0069-1

**Published:** 2018-02-09

**Authors:** Jean-Marie Volland, Arno Schintlmeister, Helena Zambalos, Siegfried Reipert, Patricija Mozetič, Salvador Espada-Hinojosa, Valentina Turk, Michael Wagner, Monika Bright

**Affiliations:** 1 Department of Limnology and Bio-Oceanography, University of Vienna, Vienna, Austria; 2 Department of Microbiology and Ecosystem Science, Research Network “Chemistry meets Microbiology” and Large-Instrument Facility for Advanced Isotope Research, University of Vienna, Vienna, Austria; 3 Cell Imaging and Ultrastructure Research (CIUS), University of Vienna, Vienna, Austria; 4 National Institute of Biology, Marine Biology Station, Piran, Slovenia

## Abstract

The giant colonial ciliate *Zoothamnium niveum* harbors a monolayer of the gammaproteobacteria *Cand*. Thiobios zoothamnicoli on its outer surface. Cultivation experiments revealed maximal growth and survival under steady flow of high oxygen and low sulfide concentrations. We aimed at directly demonstrating the sulfur-oxidizing, chemoautotrophic nature of the symbionts and at investigating putative carbon transfer from the symbiont to the ciliate host. We performed pulse-chase incubations with ^14^C- and ^13^C-labeled bicarbonate under varying environmental conditions. A combination of tissue autoradiography and nanoscale secondary ion mass spectrometry coupled with transmission electron microscopy was used to follow the fate of the radioactive and stable isotopes of carbon, respectively. We show that symbiont cells fix substantial amounts of inorganic carbon in the presence of sulfide, but also (to a lesser degree) in the absence of sulfide by utilizing internally stored sulfur. Isotope labeling patterns point to translocation of organic carbon to the host through both release of these compounds and digestion of symbiont cells. The latter mechanism is also supported by ultracytochemical detection of acid phosphatase in lysosomes and in food vacuoles of ciliate cells. Fluorescence in situ hybridization of freshly collected ciliates revealed that the vast majority of ingested microbial cells were ectosymbionts.

## Introduction

In many symbiotic mutualisms, microbial symbionts provide benefits to their eukaryote host through nourishment [1, 2]. Two principal modes of organic carbon translocation from the symbiont to the host—host digestion of symbionts and direct release of soluble organic molecules and uptake into host tissue—are well characterized in photoautotrophic [3–9] and in chemoautotrophic symbioses (see Nelson and Fisher [10]). Although all studied hosts actively digest symbionts, translocation of fixed carbon to the host through release from the symbiont has up to now been reported only for *Symbiodinium* in corals [7]; upside-down jellyfish [11]; sulfur-oxidizing, chemoautotrophic (thiotrophic) endosymbionts in solemyid and lucidid bivalves [12, 13]; and in vestimentiferans [14–16]. Much less attention has been paid to thiotrophic ectosymbiosis with the exception of the shrimp *Rimicaris*, for which transfer of fixed organic carbon from the ectosymbionts to the host was shown [17].

In mutualism, trait loss in the receiving partner may be the result of compensation of trait function by the providing partner [18, 19]. Nourished by their chemoautotrophic symbionts, compensatory trait loss is evident in some hosts that either have partly reduced (e.g., solemyid bivalves [20], stilbonematid nematodes [21]) or even entirely lost their digestive system (e.g., oligochaetes [22, 23]; vestimentiferans [24]; *Kentrophoros* ciliate [25]). Other hosts retain a fully functioning digestive system (e.g., bathymodiolin bivalves) and apparently supplement their symbiotic diet with feeding on other food sources [22].

Protists frequently form associations with bacteria [26–28], but associations with thiotrophic bacteria are known only in two protist taxa, the euglenozoans and ciliates ([25, 29–32]). In contrast to the high diversity of thiotrophic symbiont location on (ectosymbionts) or in (extracellular and intracellular endosymbionts) animal hosts, the thiotrophic symbionts of protists exclusively colonize the host extracellularly.

The giant colonial ciliate *Zoothamnium niveum* thrives in marine shallow waters on sulfide-emitting decaying plants and animal bones. The host forms a feather-shaped, sessile colony up to 1.5 cm in length. Ciliates are mostly unicellular, but the giant ciliate is a physiologically and functionally integrated, multicellular unit [33]. It is composed of a stalk and alternate branches with hundreds to thousands of individual cells with different function: the feeding microzooids, the dividing terminal zooids, and the macrozooids, which detach from the mother colony and disperse as swarmers to found new asexually produced colonies (Supplementary Figure 1, [34, 35]). Conjugation, the sexual process in ciliates, is through microgamonts that also leave the mother colony, but then fuse with a terminal zooid of another colony so that the multicellular unit is not genetically homogeneous [36, 37].

Except for the lower part of the stalk, all other host parts are covered exclusively by the gammaproteobacterial, thiotrophic ectosymbiont *Cand*. Thiobios zoothamnicoli [34, 35, 38, 39]. The ciliate colony contracts into the sulfidic layer and expands into the oxic seawater, repeatedly exposing the symbionts to fluctuating environmental conditions between fully oxic without sulfide (access to electron acceptor) and anoxic conditions with up to 300 µmol L^−1^ of sulfide (access to electron donor) [31, 40]. Symbiont cells are coccoid shaped on the upper parts of microzooids, but rod shaped on all other parts of the colony (Supplementary Figure 1C, [34]). This suggests that the coccoid-shaped symbiont on microzooids benefits from more favorable host-regulated sulfide and oxygen conditions [41]. Potential benefits of this behavior and symbiont colonization for the host are sulfide detoxification and nourishment [31].

Although this is the only reported thiotrophic symbiosis that can be cultivated over generations [41], direct evidence of the symbionts’ involvement in host nutrition has been lacking so far. Nutritional transfer through digestion of symbiont cells was hypothesized based on transmission electron microscopy (TEM) observations of symbiont-like bacteria in the cytopharynx and in digestive vacuoles of microzooids [35]. Because ciliates can directly uptake dissolved substances through active transport or pinocytosis [27], we hypothesized that the host also directly takes up organic compounds released by symbiont cells.

We used a combination of tissue autoradiography (TA) and a newly developed cryo-preparation technique [42] coupled with resin embedding and nanoscale secondary ion mass spectrometry **(**NanoSIMS) correlated with TEM to investigate the autotrophic behavior of the bacterial symbiont cells and the translocation of organic carbon to the ciliate host from populations collected from wood in the Adriatic Sea. In addition, we studied the in situ host diet using fluorescence in situ hybridization (FISH), and ultracytochemistry. We provide evidence for chemoautotrophy of the ectosymbiont using sulfide in the seawater, as well as internally stored sulfur as electron donor. We show that using bicarbonate in the seawater as a source of inorganic carbon, fixed organic carbon is rapidly released from the ectosymbiont cells to the host. Moreover, we demonstrate that the food vacuoles of the host are mainly filled by symbionts that are phagocytosed.

## Materials and methods

A detailed version of the materials and methods including the description of the sampling site is provided as supplementary material.

### ^14^C and ^13^C-bicarbonate incubations

Five colonies were incubated in normoxic seawater containing 2.5 µCi mL^−1^ NaH^14^CO_3_ (DHI®). The following incubations were carried out: (1) 12.2 µmol L^−1^ ΣH_2_S (^14^C sulfidic pulse) and (2) no sulfide (^14^C oxic pulse) for 25 min each. Aiming at depleting internal sulfur storage compounds in the symbiont cells, five additional colonies were kept in oxic conditions for 24 h prior to incubation in ^14^C-bicarbonate at oxic conditions for 25 min (24-h oxic + ^14^C oxic pulse). Two negative controls were performed: (i) colonies were killed with absolute ethanol prior to the sulfidic pulse incubation for 25 min (dead control) and (ii) colonies were incubated in 12.2 µmol L^−1^ ΣH_2_S but without adding ^14^C-bicarbonate for 25 min (natural carbon control). To follow the fate of labeled organic carbon, we performed the same sulfidic pulse incubation followed by a chase without ^14^C-bicarbonate in 12.4 µmol L^−1^ sulfide for 6 h (^14^C sulfidic pulse chase) (see Supplementary Methods and Supplementary Table 1).

We localized labeled carbon with high spatial resolution NanoSIMS analyses. Five colonies were incubated in seawater supplemented with 100 mmol L^−1^ of NaH^13^CO_3_ (Sigma-Aldrich®) and 27.1 µmol L^−1^ ΣH_2_S for 3 h and two colonies were analyzed in detail (^13^C sulfidic pulse). Another batch of five colonies was maintained in oxic seawater for 24 h prior to incubation in ^13^C-bicarbonate under oxic conditions for 3 h each (24-h oxic + ^13^C oxic pulse). As described above, a dead control and a natural carbon control were prepared (see also Supplementary Methods and Supplementary Table 1).

### Tissue autoradiography

Fixed specimens were embedded in resin and sections were processed for TA (see supplementary material). Briefly, sections were dipped in an emulsion, stored for 3 months, developed, fixed, and stained prior to light microscopic observation. The microzooids and the stalk, as well as the symbionts covering these host parts were chosen for quantitative analyses of the silver grain density (actual grain density: AGD; Supplementary Figure 2). For statistical comparisons between treatments and between cell types and stalk within the same treatment, we expressed the grain density of each area relative to a reference. As a reference, we took the average symbiont AGD. The resulting relative grain densities (RGDs) are expressed as a percentage of the reference and can be compared with each other [43].

### Correlative NanoSIMS and TEM

One colony per treatment was analyzed with NanoSIMS except for the sulfidic pulse incubation where two replicate colonies were analyzed separately (for details, see supplementary material). Briefly, specimens were cryo-fixed after chemical fixation and rapidly cryo-substituted prior to resin embedding. For correlative imaging, consecutive TEM sections were cut, placed onto slot grids, and contrasted prior to imaging with a Zeiss® Libra 120 TEM. NanoSIMS sections placed onto silicon wafer platelets were analyzed with a Cameca NS50L utilizing C^−^, CN^−^, P^−^ and S^−^ secondary ions for elemental imaging as well as C_2_^−^ secondary ions for inference of the ^13^C tracer content (displayed as ^13^C/(^12^C + ^13^C) isotope fraction, given in at%). NanoSIMS and TEM images obtained from similar analysis areas were superimposed using the GIMP® software package.

### Acid phosphatase ultracytochemistry

Three freshly collected colonies were fixed and processed for the ultracytochemical detection of acid phosphatase following Gomori’s methods ([44], for details, see supplementary material).

### 16S rRNA gene sequencing and FISH

To confirm the identity of the symbiont, 16S rRNA gene clone libraries were obtained for three colonies (see supplementary material for details). For FISH, four colonies were fixed immediately after collection in the natural environment. Colonies were embedded in LR White resin and FISH was applied on sections of entire colonies using a mix consisting of probes EUB338-I, EUB338-II, EUB338-III [45] and Arch915 [46], all labeled with Cy5 to target most bacteria and archaea. A symbiont-specific probe labeled with Cy3 was used to target the ectosymbiont *Cand*. Thiobios zoothamnicoli (ZNS196_mod, Supplementary Figure 3). We counted the symbiont-specific FISH signals in all detected digestive vacuoles and compared their numbers to those labeled only with the EUB_mix_ and Archaea probe mix to estimate the composition of the host diet (see supplementary material for detailed FISH procedure).

## Results and Discussion

### Carbon fixation and incorporation of organic carbon in the thiotrophic symbiont

**Fig. 1:**
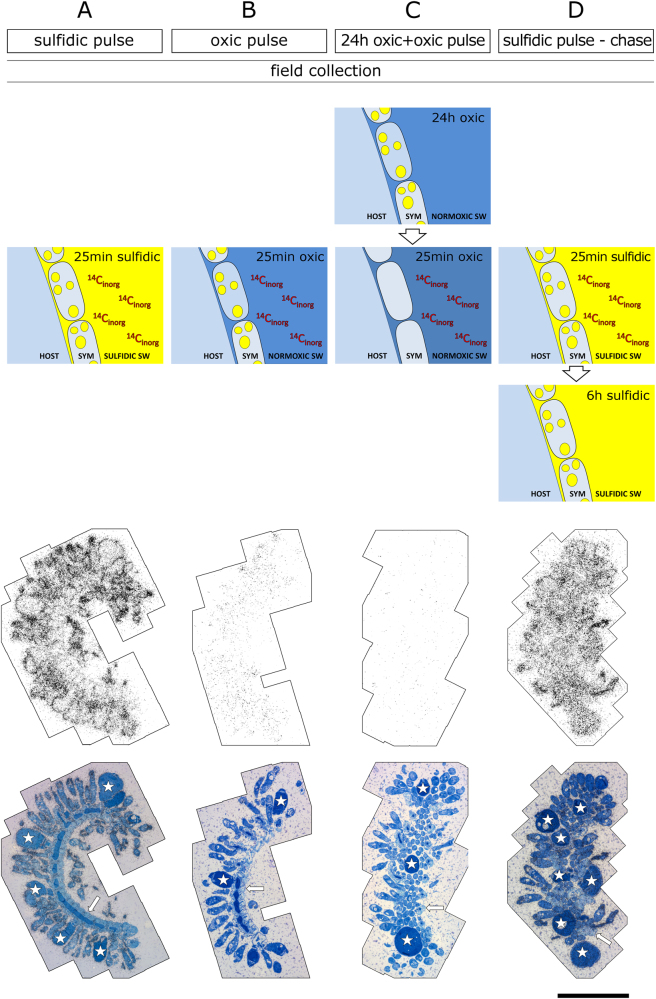
Four different ^14^C-bicarbonate incubations with the inferred cell and environmental states (top half) with corresponding autoradiographs and colony sections (bottom half). **a** Directly after collecting ciliate colonies from the environment, we conducted a pulse labeling experiment with ^14^C-bicarbonate in the presence of H_2_S. After fixation and autoradiography, colony sections were covered with silver grains, showing that ^14^C was incorporated into cellular biomass. Grains are denser in the periphery of host cells where symbionts are located. **b** After a labeling experiment with ^14^C-bicarbonate under oxic (non-sulfidic) conditions, colony autoradiographs were also covered with grains but to a lesser extent, showing that less labeled carbon was incorporated during the pulse. **c** Colonies pre-treated 24 h under oxic conditions prior to labeling with ^14^C-bicarbonate at oxic conditions did not incorporate ^14^C: no silver grains are observed on the autoradiographs except for some background signals. **d** After a pulse labeling experiment with ^14^C-bicarbonate in the presence of H_2_S as in **a**, colonies were transferred into sulfidic seawater without the radiotracer for 6 h (chase). After autoradiography, incorporated labeled carbon is detected throughout the entire colony. A detail overview on these four incubation experiments together with a description of the control incubations is given in Supplementary Table 1. Each selected autoradiograph is representative of five colonies analyzed for each treatment. Yellow color represent available reduced sulfur species (elemental S in symbionts and ΣH_2_S in the incubation media). Stars label macrozooids and arrows point to the stalk; all other cells are microzooids. Scale bar: 200 µm

**Fig. 2:**
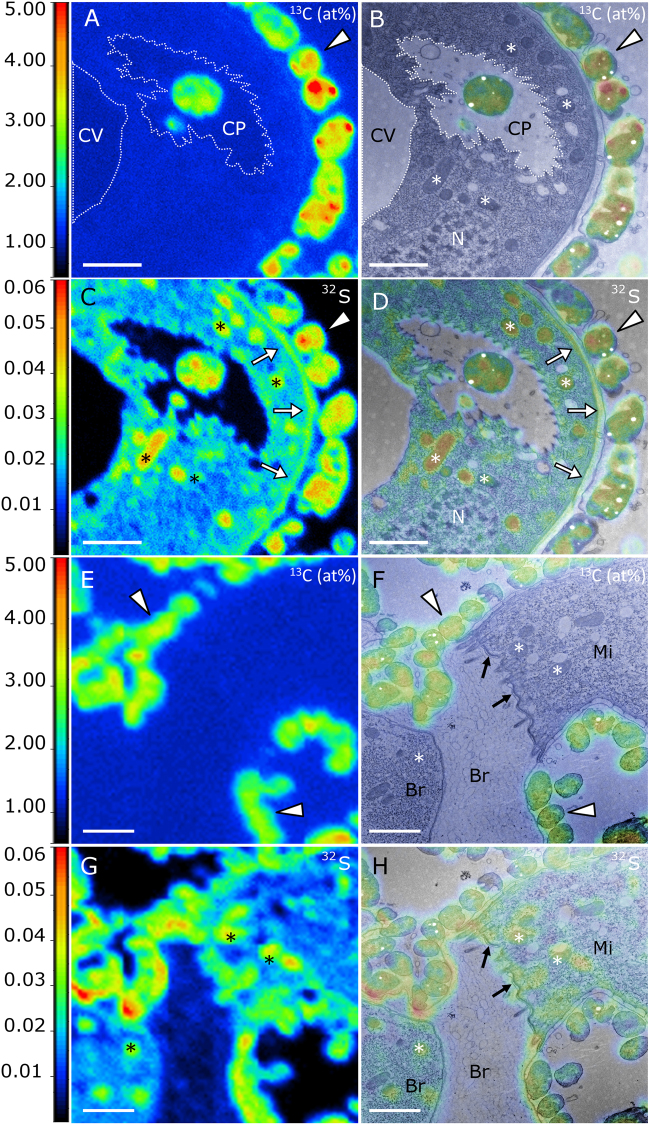
NanoSIMS/TEM correlative images after ^13^C-bicarbonate labeling in the presence of H_2_S. **a-d** Analysis of a part of a microzooid. **a** NanoSIMS visualization of the ^13^C label distribution (^13^C/(^12^C + ^13^C) isotope fraction, given in at%). The isotopically labeled carbon is mostly incorporated into symbionts (arrowhead points to one symbiont cell) but also visible within the host (c.f. data from region of interest (ROI) analysis in Fig. 3). The empty vacuole and cytopharynx (dotted line) do not show ^13^C enrichment, whereas the cytoplasm does (Fig. 3). **b** Overlay of the NanoSIMS image with the corresponding TEM micrograph showing a partial view of a microzooid surrounded by symbionts. Inside the host cell, part of the contractile vacuole (CV) is visible, and a ^13^C-rich symbiont-like bacterium is recognizable in the cytopharynx (CP). Many mitochondria are visible, some are labeled with asterisks (*). Region “N” refers to a macronucleus. **c** NanoSIMS visualization of the relative sulfur content as inferred from the C^−^ normalized ^32^S^−^ secondary ion signal intensity (see section Materials and methods in the supplemental material for details). **d** TEM/NanoSIMS overlay showing sulfur being more concentrated in symbionts, mitochondria, and cortex of host (white arrows). **e-h** Detail of the basal part of a microzooid connected to a branch. **e** NanoSIMS visualization of the ^13^C label distribution (^13^C/(^12^C + ^13^C) isotope fraction, given in at%). **f** Overlay of the NanoSIMS image with the corresponding TEM micrograph showing the connection (black arrows) between the microzooid (Mi) and the branch (Br). Enriched symbionts (arrowheads) cover both structures. Mitochondria (*) are visible in both the microzooid and the branch. **g** NanoSIMS visualization of the relative sulfur content. **h** Overlay of the NanoSIMS inferred sulfur distribution with the corresponding TEM micrograph. Asterisks label mitochondria. Scale bars: **a**-**h** 2 µm

**Fig. 3:**
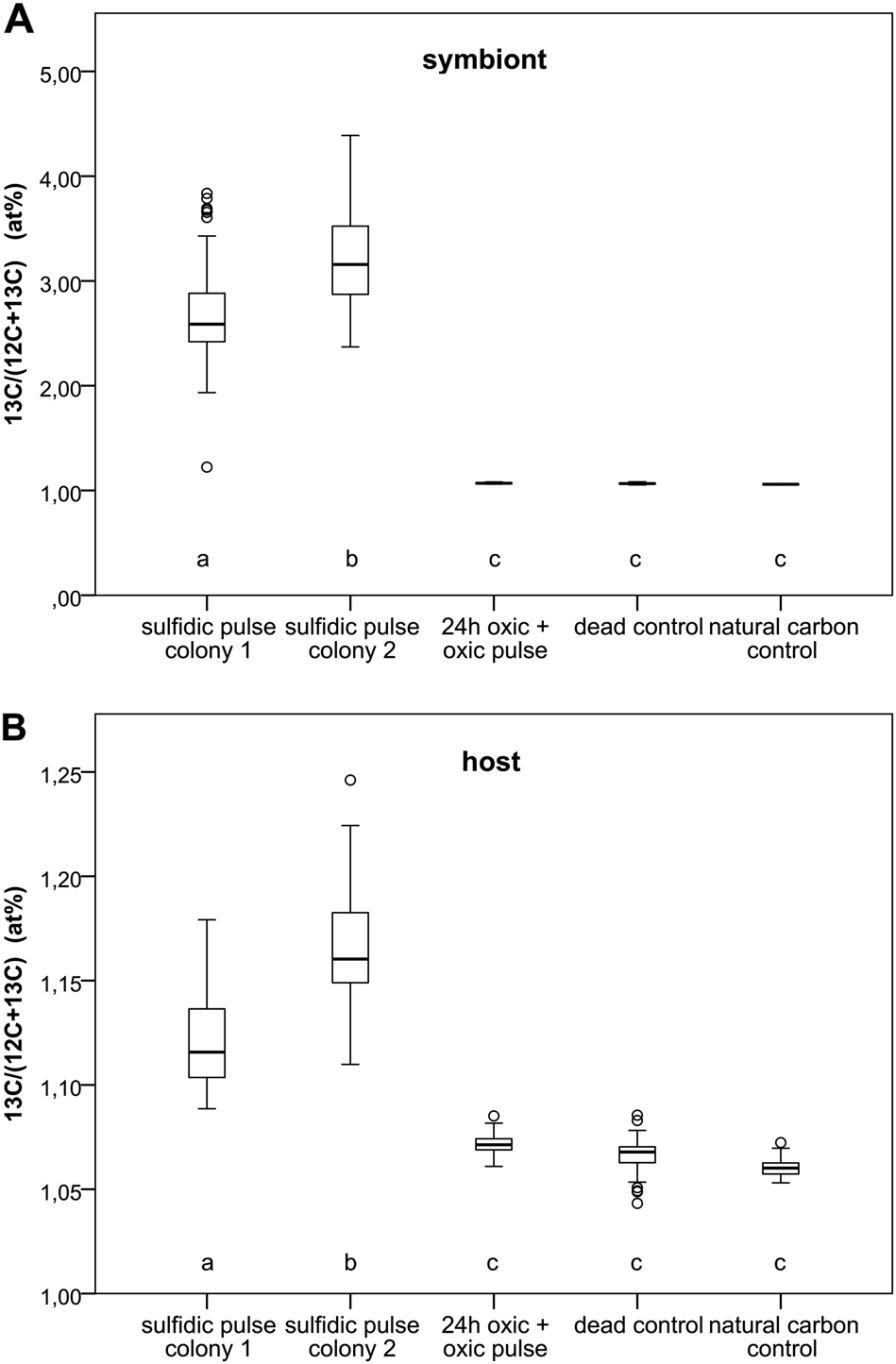
NanoSIMS ROI analysis of the ^13^C label content in symbionts and host after the different ^13^C-bicarbonate incubations. **a** ^13^C isotope fraction within the symbiont after the sulfidic incubation, the 24-h oxic + oxic incubation and the two control incubations. Symbionts significantly incorporated ^13^C when incubated in the presence of sulfide (result of Scheffe test is shown with letters a, b, and c, alpha = 0.001). Colonies first treated 24 h in oxic conditions and then incubated in oxic conditions did not incorporate labeled carbon and resemble the natural abundance of ^13^C (c.f. values from the two controls). **b** ^13^C isotope fraction within the host after the same incubations. Significant host ^13^C enrichment is detected after the sulfidic pulse (result of Scheffe test is shown with letters a, b, and c, alpha = 0.001). The 24-h oxic + oxic incubation did not lead to significant ^13^C enrichment. Letters shared in common between groups indicate no significant difference

Key genes for autotrophic carbon fixation and sulfur metabolism found in the symbiont suggested a thiotrophic metabolism [38, 47]. Consistently, previous cultivation experiments revealed highest host and symbiont fitness under low sulfide/high oxygen conditions [41], but direct proof of a thiotrophic symbiont lifestyle was lacking. To investigate the autotrophic behavior of the symbiont under these optimal conditions, we performed short labeling experiments with ^14^C- and ^13^C-bicarbonate in the presence of H_2_S for 25 min and 3 h, respectively, and followed carbon incorporation with TA and NanoSIMS. Autoradiographs revealed high numbers of silver grains over symbiont cells (*N* = 5, AGD 23.06, interquartile range (IQR) 15.79–25.67; Fig. 1a and Table 1). Similarly, NanoSIMS isotope analysis and correlative TEM micrographs at higher resolution clearly showed that the symbiont was the site of incorporation (*N* = 291 symbiont cells, ^13^C isotope fraction 2.68 at%, IQR 2.45–3.04; Figs. 2, 3a). In contrast, dead colonies incubated under identical conditions showed no incorporation (*N* = 3 colonies, AGD 0.00, IQR 0.00–0.00; *N* = 50 symbiont cells, ^13^C isotope fraction 1.07 at%, IQR 1.06–1.07) and the same results were obtained for living colonies incubated without added bicarbonate (*N *= 3 colonies, AGD 0.02, IQR 0.01–0.03; *N* = 50 symbiont cells, ^13^C isotope fraction 1.06 at%, IQR 1.06–1.06; Fig. 3 and Tables 1 and 2). This strongly suggests that the symbiont cells rapidly fix inorganic carbon in the presence of sulfide, similar to certain other thiotrophic symbionts [48–53].

**Table 1:** Summary of the analyses of the autoradiographs

	Sulfidic pulse			Oxic pulse			24-h oxic + oxic pulse		Sulfidic pulse-chase		Wilcoxon–Mann–Whitney test	
	AGD	RGD		AGD	RGD		AGD	RGD	AGD	RGD	sp vs op	sp vs spc
Microzooid symbiont	27.66	107.78 **		3.63	112.88 **		0.12	–	21.39	103.36	nd	nd
	(16.97–28.93)	(107.53–110.50)		(3.29–3.67)	(102.94–117.83)		(0.12–0.13)	–	(16.06–27.34)	(101.36–103.83)		
Stalk symbiont	9.20	51.75		2.03	68.30		0.02	–	16.63	80.97	nd	nd
	(8.17–10.65)	(31.61–69.82)		(2.00–3.58)	(61.81–68.76)		(0.01–0.08)	–	(14.22–21.32)	(79.31–90.69)		
**Total symbiont**	**23.06**	**100.00**		**2.97**	**100.00**		**0.09**	**–**	**19.79**	**100.00**	**nd**	**nd**
	(15.79–25.67)	(100–100)		(2.91–3.63)	(100–100)		(0.08–0.11)	-	(15.96–26.33)	(100–100)		
Microzooid host	10.44	44.36		1.05	32.65		0.07	–	12.65	63.89	nd	*
	(4.69–11.39)	(35.62–45.25)		(0.71–1.18)	(32.53–35.56)		(0.06–0.10)	–	(9.36–21.37)	(63.12–69.60)		
Stalk host	4.17	19.38		0.63	21.61		0.03	–	9.13	50.53	nd	**
	(0.46–5.60)	(2.89–21.81)		(0.43–1.25)	(14.51–24.57)		(0.03–0.03)	–	(8.79–11.56)	(44.40–67.85)		
**Total host**	**4.56**	**24.90**		**0.68**	**20.00**		**0.09**	**–**	**12.92**	**65.26**	**nd**	***
	(2.31–6.39)	(9.99–33.75)		(0.59–1.10)	(19.04–30.29)		(0.04–0.11)	–	(10.73–18.38)	(61.59–69.8)		

For each incubation, the medians of the actual grain density (AGD) and relative grain density (RGD) of five replicates are shown together with the interquartile range of the data (Q25–Q75). The RGDs are the grain counts, which are set as a proportion of the overall symbiont grain count. All comparisons were then performed on the RGDs. Within a particular treatment, comparisons of the symbionts covering the microzooid and stalk areas as well as the respective host areas were performed using Wilcoxon–Mann–Whitney significance testing. This test was also performed to compare the sulfidic pulse and the oxic pulse (sp vs op), as well as the sulfidic pulse and the sulfidic pulse chase incubations (sp vs spc). Bonferroni correction was then applied

*nd* not different

*p  < 0.05; **p <  0.01; ***p  < 0.001

**Table 2:** Summary of the ROI analysis of NanoSIMS ^13^C label distribution images

	Sulfidic pulse	24-h oxic + oxic pulse	Dead control	Natural carbon control
Microzooid symbiont	3.05 ***	1.07	1.07	1.06
	(2.69–3.28)	(1.07–1.07)	(1.06–1.07)	(1.06–1.06)
	*n* = 141	*n* = 50	*n* = 50	*n* = 50
Stalk symbiont	2.51	–	–	–
	(2.36–2.68)	–	–	–
	*n* = 150	–	–	–
**Total symbiont**	**2.68 a**	**1.07 b**	**1.07 b**	**1.06 b**
	(2.45–3.04)	(1.07–1.07)	(1.06–1.07)	(1.06–1.06)
	*n* = 291	*n* = 50	*n* = 50	*n* = 50
Microzooid host	1.15 ***	1.07	1.07	1.06
	(1.13–1.16)	(1.07–1.07)	(1.06–1.07)	(1.06–1.06)
	*n* = 100	*n* = 50	*n* = 50	*n* = 50
Stalk host	1.10	–	–	–
	(1.10–1.11)	–	–	–
	*n* = 50	–	–	–
**Total host**	**1.13 a**	**1.07 b**	**1.07 b**	**1.06 b**
	(1.11–1.15)	(1.07–1.07)	(1.06–1.07)	(1.06–1.06)
	*n* = 150	*n* = 50	*n* = 50	*n* = 50

For the sulfidic pulse and the 24-h oxic + oxic pulse, as well as for the two control experiments, the median of the ^13^C isotope fraction (^13^C/(^12^C + ^13^C), given in at%) is shown together with the interquartile range of the individual data points (Q25-Q75). (*n*) refers to the number of replicates analyzed within each treatment. For the symbiont, one replicate is one ROI drawn around one individual symbiont. For the host, the replicates are randomly selected ROIs within host cytoplasm. The Scheffe test (alpha = 0.05) was used to compare the ^13^C label content in the symbiont and host after the different treatments. The result of the Scheffe test is given with lowercase letters “a” and “b”, letters shared in common between groups indicate no significant difference. The Wilcoxon–Mann–Whitney test was performed to compare the microzooid and stalk area after the sulfidic pulse for both symbiont and host ^13^C enrichment.The results of this test is shown with asterisks.

***p  < 0.001

Because the symbiont cells store elemental sulfur in membrane-bound vesicles [54] and oxygen consumption measurements suggested that the symbionts completely utilize intracellularly stored sulfur in the absence of sulfide within 4 h [40], we hypothesized that the internal sulfur acts as electron donor for the thiotrophic metabolism, providing the energy needed for carbon fixation under oxic conditions. Therefore, we performed a short ^14^C-bicarbonate-labeling experiment under oxic conditions in the absence of H_2_S and found an approximately eightfold lower median AGD over symbiont cells (*N* = 5; AGD 2.97, IQR 2.91–3.63), than after the sulfidic pulse (Fig. 1b and Table 1), but significantly higher than the AGD of the controls. This points to the symbionts using elemental sulfur as an electron donor, gaining energy through sulfur oxidation and fixing carbon under oxic conditions, but to a lesser degree than when external sulfide was provided via the seawater.

To confirm that the internal sulfur storage indeed lasts only briefly [40], we kept colonies for 24 h in oxic seawater prior to labeling with ^14^C- and ^13^C-bicarbonate under oxic conditions for 25 min and 3 h, respectively. We hypothesized that after prolonged oxic conditions, sulfur was depleted and therefore carbon fixation ceased. Indeed, after this treatment the median AGD over symbiont cells (*N* = 5; AGD 0.09, IQR 0.08–0.11; Fig. 1c and Table 1), as well as the ^13^C content in symbiont cells (*N* = 50, ^13^C isotope fraction 1.07 at%, IQR 1.07–1.07%; Fig. 3 and Table 2) were in the range of the negative controls. These results indicate that chemoautotrophy has ceased within 24 h, most likely due to the lack of an electron donor (absence of an external sulfide source in oxic seawater and the depletion of sulfur stored in the symbionts).

**Fig. 4:**
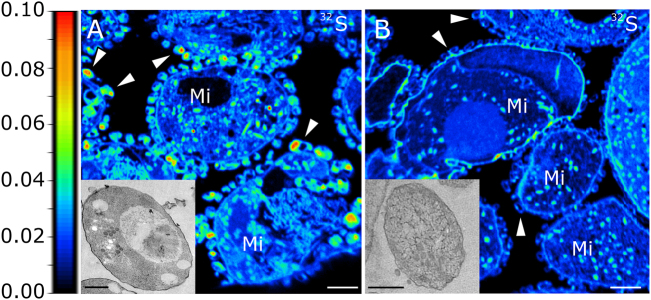
Effect of the 24-h oxic treatment on the symbiont internal sulfur storage. The color scale indicates the C^−^ normalized ^32^S^−^ secondary ion signal intensity. **a** NanoSIMS visualization of the relative sulfur content in a colony from the sulfidic pulse. Internal sulfur storage appears as sulfur hot spots in the symbionts (arrowheads) surrounding the microzooids (Mi) host cells. **b** NanoSIMS visualization of the relative sulfur content in the colony treated 24 h in oxic conditions before the oxic labeling experiment. The symbionts no longer show sulfur-rich regions. Representative TEM images of symbiont cells of corresponding treatments are given in the respective inserts. Symbionts show various electron-lucent vesicles identified by NanoSIMS as sulfur vesicles **a**, which are lacking after the 24-h incubation in oxic conditions **b**. NanoSIMS scale bars: 4 µm; TEM scale bars: 500 nm

NanoSIMS imaging revealed sulfur-rich areas in the cytoplasm of microzooids. The correlative TEM images showed that these areas corresponded to mitochondria (Fig. 2), known to have abundant disulfide bonds located in the inter-membrane space and membrane proteins [55]. Correlative TEM imaging of symbiont cells showed membrane-bound vesicles, which in successive sections analyzed by NanoSIMS exhibited high sulfur signals in restricted, roundish areas of the symbiont cells (Fig. 4). Thus, NanoSIMS imaging confirmed the presence of sulfur in the symbiont cells after incubations in sulfidic seawater (^32^S^−^/C^−^ signal intensity ratio in symbiont cells 0.028, IQR 0.025–0.031, for details, see supplementary material). In contrast, after 24 h in oxic seawater, only a few and very small sulfur vesicles were observed in TEM and the relative amount of sulfur in the symbiont cells was significantly reduced (^32^S^−^/C^−^ signal intensity ratio 0.016, IQR 0.012–0.020; Fig. 4). These results corroborate the depletion of most of the internally stored sulfur within 24 h under oxic conditions.

The ^13^C contents measured within individual symbionts on two separate colonies kept at sulfidic conditions for 3 h were significantly different (Wilcoxon–Mann–Whitney test, *p*-value 4×10^−18^) and ^13^C isotope fractions varied between 2.42 and 4.54 at% (*N* = 50) and 1.22 and 3.84 at% (*N* = 241), respectively (Fig. 3). The sulfur-related signal intensities in these cells were ^32^S^−^/C^−^ 0.031, IQR 0.027–0.035 and ^32^S^−^/C^−^ 0.028, IQR 0.020–0.031, respectively. This demonstrates that overall oxic seawater supplemented by 27.1 µmol L^−1^ sulfide (and even 12.2 µmol L^−1^ sulfide when taking ^14^C experiments into account) is sufficient to fuel an active thiotrophic metabolism maintaining sulfur storage. This is consistent with the experimental optimal conditions in oxic seawater supplemented with 3–33 μmol L^−1^ sulfide [41]. Note that sulfide concentrations from 0.1 to 100 µmol L^−1^ were recorded from the sunken wood surface colonized by *Z. niveum* [56]. Nonetheless, the variability in metabolism was apparently high among colonies. Metabolic activity was variable among colonies and among the 141 investigated individual symbiont cells located on microzooids next to each other. A positive correlation of ^13^C content and sulfur suggests that under sulfidic conditions the more carbon is fixed the more sulfur is also stored (Supplementary Figure 4).

### Symbiont phenotypic plasticity and carbon incorporation

To investigate whether symbionts located on different parts of the colony incorporate carbon to different degrees, we compared the RGDs of symbiont populations covering the microzooids and the stalk. The RGDs of the mixed symbiont populations on the microzooids (with the upper part covered with cocci and the lower part covered with rods; [34]) were significantly higher than the values of the rods on stalks [34] after both the oxic and the sulfidic pulses (Table 1, Wilcoxon–Mann–Whitney test, *p-*value 0.008). Indeed, precise analyses of individual symbiont cells with NanoSIMS confirmed that ^13^C enrichment in symbiont cells covering microzooids (*N* = 141 symbiont cells; 3.05 at%, IQR 2.69–3.28) was significantly higher than that of cells on stalks (*N* = 150 symbiont cells; median 2.51 at%, IQR 2.36–2.68) (Table 2) although they were not significantly different in size in the analyzed sections (Wilcoxon–Mann–Whitney test,* p*-value 0.385).

Overall, the symbiont cells on microzooids incorporated more labeled carbon than the rods on other colony parts. These results are in agreement with the ciliate behavior creating fine-scale differences in oxygen and sulfide concentrations along the microzooids [41]. The oral cilia of the microzooids resume beating as soon as the colony expands into the oxic seawater after dipping into the sulfidic layer through contraction of the spasmoneme, a special protein located throughout the stalk and the branches [57, 58]. The larger coccoid-shaped symbiont cells therefore were proposed to receive a mix of chemicals more favorable for chemoautotrophy than the smaller rods on all other parts of the colony [41]. When colonies were cultivated under steady oxygen and sulfide concentrations, the entire symbiont population grew as rods [41]. These results suggest that differences in previously described morphotypes are due to differences in chemoautotrophic rates leading to higher carbon incorporation in coccoid versus rod-shaped cells.

### Organic carbon translocation through release and uptake

Carbon fixation and release of organic carbon into the surrounding host tissue is virtually concomitant in endosymbionts of *Riftia pachyptila* [16]. We therefore asked whether potential release from the ectosymbionts, which are attached to the ciliate host on one side only, occurs and leads to uptake by the host. Ciliates can directly take up dissolved organic carbon [59–63]. Some can even grow in axenic, nutrient-rich media without added prey [64, 65]. Our experiments enable differentiating between both nutrient translocation modes. Release of nutrients by the symbiont and subsequent uptake by the host should occur much faster than nutrient transfer via symbiont digestion by the host. This should enable the detection of the isotope label in the host after an incubation with ^14^C-bicarbonate that is short enough to exclude digestion. Minimal time for digestion in ciliates is 30 min, with maxima of up to 5 h [66, 67].

^14^C-bicarbonate labeling for 25 min resulted in labeled carbon in host tissue after sulfidic (*N* = 5 colonies; AGD 4.56, IQR 2.31–6.39) and oxic incubations (*N* = 5 colonies; AGD 0.68, IQR 0.59–1.10; Figs. 1a, b, Table 1). We found no significant differences between stalk and microzooids (Table 1). Rather than symbionts fixing and incorporating inorganic carbon followed by host feeding and digestion of labeled symbiont cells within 25 min, we suggest that labeled organic carbon compounds produced by the symbiont cells are released, directly taken up by the host and incorporated into its tissue. Note that these experiments revealed some labeled microbial cells in food vacuoles, but we excluded them from statistical analyses because they were clearly not yet digested and incorporated into host tissue. Moreover, in the colonial ciliate, phagocytosis is restricted to microzooids [35], but RGDs over the non-feeding stalk was not significantly different to that over feeding microzooids. Therefore, our results are consistent with the host taking up released carbon from the ectosymbionts. Direct release of nutrients by the symbiont and host uptake was also demonstrated in *Solemya reidi* (with >45% of the fixed carbon being translocated to the bivalve; [12]) and in *Riftia pachyptila* (with 15.3 ± 4.5% RGD in tubeworm tissue after ^14^C-bicarbonate 15-min pulse incubation; [16]). The RGDs in the ciliate host represent the host label as a percentage of the symbiont label. Microzooids, which represent most of the host biomass, have RGDs of 44.36% (IQR 35.62–45.25) after the sulfidic pulse and 32.65% (IQR 32.53–35.56) after the oxic pulse (Table 1). The ciliate uptake of released organic compounds is in the same order of magnitude as reported for the bivalve and vestimentiferan hosts.

In some symbioses, the host enhances the release of organic compounds from its symbiont. Evidence of the host influencing the rate of release was found in corals by comparing the amount of released compounds in host-associated and free-living *Symbiodinum* cells [68–70]. Because we have not detected a free-living symbiont population, such a comparison of release between host-associated and free-living symbiont populations is not possible in our system. Therefore, we compared the relative amount of label in hosts after oxic and sulfidic pulse incubations with labeled bicarbonate. The hypothesis is that, under host control, uptake of leaked organic carbon should be higher when symbionts fix less carbon under oxic conditions compared with higher fixation rates under sulfidic conditions. Alternatively, under symbiont control, a lower release should be found under oxic versus sulfidic conditions. The RGDs represent the host label expressed as a percentage of the average symbiont label in the colony, therefore showing the proportion of symbiont-fixed carbon transferred to the host through release during the pulse. Remarkably, no significant differences were observed in RGDs of host tissue between sulfidic (*N* = 5 colonies; RGD 24.90%, IQR 9.99–33.75) and oxic (*N* = 5 colonies; RGD 20.00%, IQR 19.04–30.29) incubations. These results indicate a stable amount of released carbon correlated to the amount of fixed carbon governed by environmental conditions (which may be more or less favorable for chemosynthesis). Therefore, in contrast to corals, the ciliate host is apparently unable to enhance the release of fixed carbon from the symbiont under less favorable oxic conditions. Similarly, the symbiont apparently is also unable to actively reduce the release. About one order of magnitude lower AGDs in symbionts and host under less favorable oxic conditions compared with more favorable sulfidic conditions are consistent with this interpretation (Table 1).

### Organic carbon translocation by symbiont cells digestion

The symbiont cells were highly labeled with ^14^C at the end of the sulfidic pulse, therefore we transferred some colonies to sulfidic seawater without ^14^C-bicarbonate for 6 h of chase in order to follow the fate of labeled carbon incorporated in the symbiont. To investigate whether symbiont cells labeled during the 25-min pulse continued to release labeled carbon in the 6-h chase with no ^14^C-bicarbonate available, we compared the AGDs of symbionts between the sulfidic pulse and the sulfidic pulse-chase and found no significant differences (Table 1, Wilcoxon–Mann–Whitney test, *p*-value 0.841). We conclude that no further major leakage of labeled organic compounds occurred during the chase time. At the same time, however, the host RGD significantly increased 2.6-fold after the 6-h chase (Table 1). As the only source of labeled carbon for the ciliate cells was labeled symbiont cells, this observation is consistent with digestion of the symbiont, similar to pulse chase experiments in bathymodiolin mussels and vestimentiferans [16, 71].

**Fig. 5:**
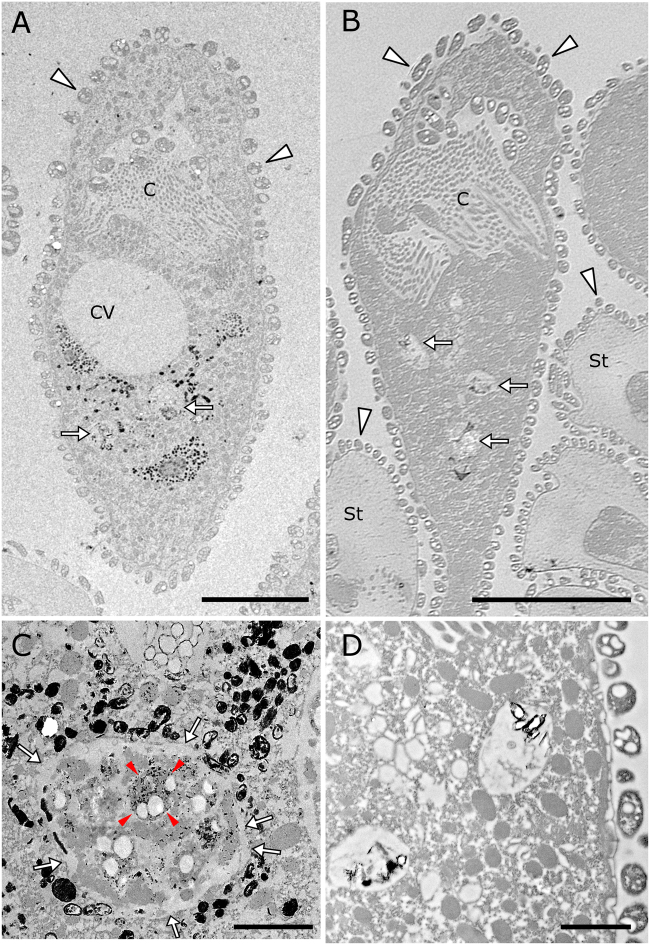
Cytochemical detection of the digestive enzyme acid phosphatase in microzooids. **a** Survey over one entire microzooid after immersion in the reactive medium to detect acid phosphatase activity. Two digestive vacuoles (arrows) are surrounded by electron-dense precipitates resulting from acid phosphatase activity. **b** Survey over one entire microzooid from the negative control in which the analogous compound of the enzyme’s substrate was omitted. No electron-dense precipitates are visible around digestive vacuoles (arrows). **c** Detail image of one digestive vacuole from the reactive medium. The borders of the vacuole are shown by white arrows. Electron-dense precipitates mostly located in small vacuoles identified as lysosomes indicate enzyme activity. The lysosomes surround the digestive vacuoles, where symbiont-like bacteria in various states of digestion are observed. One of the symbiont-like bacteria is shown by red arrowheads. **d** Detail image of two digestive vacuoles from the negative control. Triangles point to symbionts. C cilia, CV contractile vacuoles, St stalk. Scale bars: **a**, **b** 10 µm; **c**, **d** 2 µm

Phagotrophy in microzooids was already hypothesized based on ultrastructural observations of a fully developed digestive system and symbiont-like bacteria in the cytopharynx and inside digestive vacuoles [35]. In ciliates, ingested organisms observed in digestion vacuoles are not necessarily digested [72]. We therefore sought to detect acid phosphatase, an intracellular digestion marker, ultracytochemically [73]. Acid phosphate is present in ciliate lysosomes and food vacuoles, and in situ detection of this enzyme has been commonly used to highlight digestion of food [72, 74, 75]. Enzymatic activity resulting in electron-dense precipitates was detected in microzooids in small vesicles, identified as lysosomes, and in large digestive vacuoles frequently containing symbiont-like bacterial cells in various stages of degradation (Fig. 5). The cytochemical detection of this enzyme allowed unequivocal identification of the digestive process in the ciliate microzooids (Fig. 5). Based on the distribution of signal, we propose that the enzyme is produced by the ciliate cells in lysosomes and secreted into the food vacuoles for digestion (rather than ingested bacteria actively secreting acid phosphatase and undergoing autolysis) [76]. Similar ultrastructural observations and/or cytoenzymatic investigations pointed to digestion of endosymbionts in the gills of bathymodiolin mussels [71, 77, 78] and lucinid clams [79, 80], as well as in the trophosome of vestimentiferans [16].

**Fig. 6:**
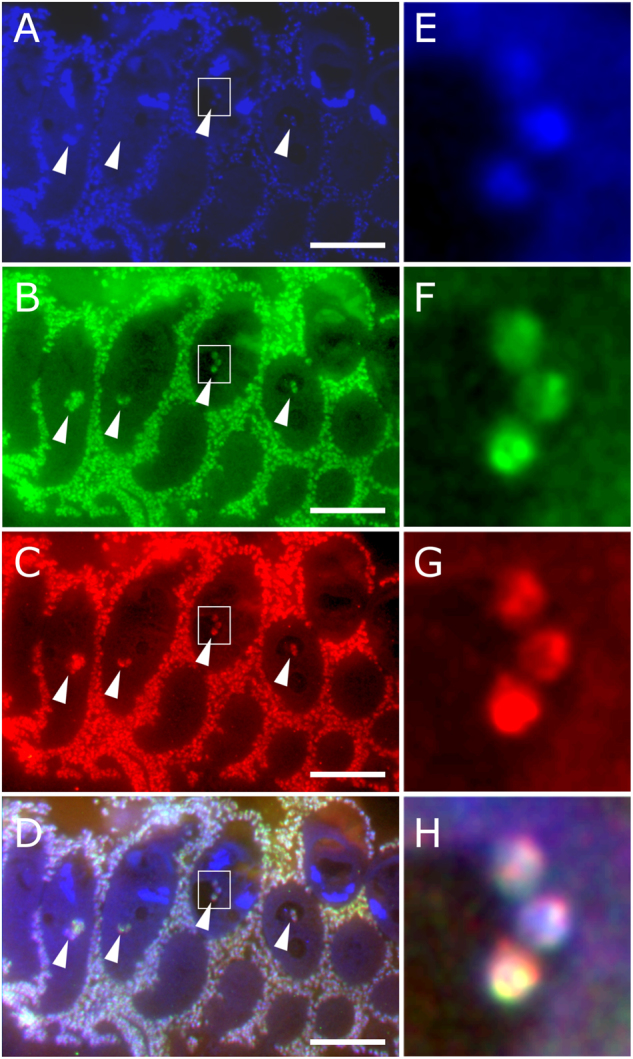
FISH micrographs of a colony semi-thin section. **a** DAPI staining. **b** Cy5 probe mix of EUB I-III and Arch915. **c** Cy3 *Cand*. Thiobios zoothamnicoli Piran-population-specific probe. **d** Overlay. All ectosymbionts surrounding the host cells are labeled with both the bacterial and the symbiont-specific probe. Four microzooids are present with digestive vacuoles (arrowheads) containing ingested symbionts. Detail of one of the digestive vacuoles is given on the right of each micrograph **e**-**h**. Scale bars: **a**-**d** 20 µm

To unambiguously identify the ectosymbiont within the digestive vacuoles, we sequenced the 16S rRNA gene of the symbiont population from Slovenia. Interestingly, the obtained 16S rRNA gene had a single mismatch in the target region of the FISH probe ZNS196 [39] that was used for identify populations from Corsica, Belize [38], and Japan [47]. We therefore modified the probe sequence in order to obtain a fully complementary FISH probe for this symbiont population. Epifluorescence microscopy revealed no difference in signal brightness between the symbiont specific and the bacterial/archaeal probe mix. Subsequently, we counted the symbiont-specific FISH signals and compared their numbers to those labeled only with the bacterial/archaeal probe mix to estimate the composition of the ciliate cells diet in four freshly collected colonies from the environment. In all, 53 digestive vacuoles were detected in 491 microzooids and analyzed. The symbiont cells highly dominated the food vacuole population (83.3–97.2% of total microbes) (Fig. 6, Supplementary Table 2). Our results indicate that mainly the ectosymbionts, and to a lesser degree other microbes from the surrounding seawater, are ingested and ultimately digested. Considering that the ciliate is also capable of filter feeding on the free-living, non-symbiotic microbes in the seawater, our results emphasize the important role of the symbiont for the host’s diet. In this context, the rapid colony contraction could play a role in the detachment of the symbiont cells from the plasma membrane of the outer host cell surface, a hypothesis that remains to be tested in the future [41, 58].

## Conclusion

Recently we proposed that the *Zoothamnium niveum*—*Cand*. Thiobios zoothamnicoli association is a byproduct mutualism in which the symbiont cells benefit from the host behavior by gaining access to electron donors and acceptors for chemosynthesis and the host benefits from released organic carbon that the symbiont cells produce [31]. Here we provide experimental evidence of symbiont carbon fixation under various environmental conditions and suggest that the release of fixed carbon to the host is indeed a byproduct benefit, controlled neither by the symbiont nor by the host. In addition, active host cells’ digestion of mainly symbiont cells contributes considerably to the host’s diet and may also help control the population density on the host. Such control might be important for the host to avoid being overgrown by its symbiont and suffocate. The presence of a perfect symbiont monolayer on the ciliate surface indicates a tight coupling of host and symbiont growth [31] fueled by the autotrophic behavior of the symbiont. How this mutualism is maintained over a wide range of environmental conditions in situ and how the shared fixed carbon and host digestion are finely tuned remain to be studied.

## Electronic supplementary material

Supplemental Material
