# Supplementary material for: NanoSIMS and tissue autoradiography reveal symbiont carbon fixation and organic carbon transfer to giant ciliate host
Source: ISME J. 2018 Feb 9;12(3):714–27. doi: 10.1038/s41396-018-0069-1 (PMC5854253; doi:10.1038/s41396-018-0069-1)
Supplement: Supplementary file 1 — Supplemental Material [file 41396_2018_69_MOESM1_ESM.docx]

SUPPLEMENTARY MATERIAL

Supplementary Materials and Methods:

**Sample collection**

*Zoothamnium niveum* colonies were collected from degrading wood pieces between 0.5 to 1 m depth in the estuarine canal of Sv. Jernej, Portorož, Northern Adriatic Sea (45°29'52.7"N, 13°35'36.7"E) in August and October 2012 and in July 2014.

**^14^C and ^13^C bicarbonate incubations**

Immediately after collection, five colonies each were incubated in closed embryo dishes in 2.2 mL of 0.2 µm filtered seawater containing 2.5 µCi mL^-1^ NaH^14^CO_3_ (DHI®). The incubation media were prepared by adding 275 µL of 2.4 mmol L^-1^ NaH^14^CO_3_ containing 20 µCi mL^-1^ to 1.925 mL of seawater. The oxygen concentration, recorded at the beginning of each incubation using a PreSenS® Flow-through Cell FTC-PSt3 probe, varied between 240.0 and 264.6 µmol L^-1^ among the different incubations. Sulfide concentration (ΣH_2_S, i.e., sum of all forms of dissolved sulfide (Le Bris *et al.,* 2006)) was measured using a quantitative colorimetric assay (Cline, 1969). After incubations the specimens were rinsed in successive sterile seawater baths, fixed in a modified Trump’s fixative (McDowell and Trump 1976) and stored in the fixative until processing two months later.

To localize isotopically labeled, fixed carbon in the symbiont cells and the host with high spatial resolution, NanoSIMS analyses were performed with colonies incubated in NaH^13^CO_3_ (Sigma-Aldrich®). Five colonies were incubated in seawater supplemented with 100 mmol L^-1^ of NaH^13^CO_3_ with 27.1 µmol L^-1^ ƩH_2_S for 3 h each and two colonies were analyzed in detail (**^13^C sulfidic pulse**). Another batch of 5 colonies was maintained in oxic seawater for 24 h prior to incubation in ^13^C bicarbonate under oxic conditions for 3 h each (**24h oxic + ^13^C oxic pulse**). As described above a **dead control** and a **natural carbon control** were prepared (see also Supplementary Table 1). After the pulse incubations the specimens were washed in successive sterile seawater baths, fixed in the modified Trump’s fixative as described above and stored in the fixative until processing five months later.

**Tissue autoradiography**

Specimens fixed in a modified Trump’s fixative (2.5% glutaraldehyde, 2% paraformaldehyde in sodium cacodylate 0.1 mol L^-1^; 1100 mOsm L^-1^; pH 7.2) were dehydrated with ethanol and embedded in medium-grade LR-White resin (Electron Microscopy Science®). Polymerization was performed under nitrogen atmosphere at 40°C for three days. Semithin sections (0.5 µm) were cut on a Leica® EM UC7 and mounted on gelatin-coated slides. Slides were hand dipped in Kodak® NTB liquid emulsion (43°C), air dried for 30 min, and stored at 4°C for 3 months exposure (Roger, 1979). The exposure time was determined empirically with a time series to evaluate the optimum signal-to-background ratio (Roger, 1979). The slides were developed with Carestream® Kodak® developer, fixed in Carestream® Kodak® fixer according to manufacturer’s instructions and stained with a solution of 0.25% azure II, 0.25% methylene blue, 0.25% toluidine blue in 0.25% sodium borate (Erikson and Fisher, 1990). All steps from the emulsion dipping to the fixation were performed in complete darkness. The slides were then observed on a Zeiss® Axio Imager A1 light microscope. Multiple micrographs with overlapping fields were taken using a 100x objective and bright field illumination to cover each entire colony in the focus plane of the section as well as in the focus plane of the silver grains above the section. The micrographs were then assembled with the image stitching program MICE® to produce two composite pictures of each entire section.

Symbionts covering the microzooids and the stalk as well as the respective host cell type and structures were selected, and the silver grains on them were quantified as number of black pixels over defined areas in percent (Actual Grain Density: AGD). AGDs were calculated over the entire section for each cell and tissue type using the GNU Image Manipulation Program (GIMP®). As background we took the empty resin area directly next to the symbionts, therefore including not only the few unspecific grains formed above the empty resin sections, but also the scattered signal from symbionts. The background AGD was then subtracted from all other analyzed areas. Therefore, any remaining grain counts in the host were not biased by the scattered signal from the symbiont and were interpreted as incorporated organic carbon in host cells and tissues. For statistical comparisons between treatments and between cell types and tissues within the same treatment, we expressed the grain density of each area relative to a reference. As a reference we took the average symbiont AGD. The resulting Relative Grain Densities (RGDs) are expressed as a percentage of the reference and can be compared with each other (Roger, 1979). To control for chemography, the three colonies of the natural abundance control were fixed and processed: no chemography was detected. To control for unspecific ^14^C enrichment of the samples (by binding of ^14^C-bicarbonate), the three colonies of the dead control were fixed and processed similarly and no label was observed. Data are presented as median and interquartile range (IQR), which were evaluated by applying the "Tukey's hinges" quartile method (Tukey 1977).

**Correlative nanoscale secondary ion mass spectrometry (NanoSIMS) and transmission electron microscopy (TEM)**

Five months after the chemical fixation, specimens were plunge frozen in liquid propane at -180°C, cryo-substituted rapidly in acetone containing 1% w/v osmium tetroxide using the new agitation module described in Goldammer et al. (2016) and embedded in Agar Low Viscosity Resin (Agar Scientific®). Polymerization was carried out at 60°C for three days. For correlative imaging, consecutive sections were cut from the resin blocks, with thicknesses alternating between semithin (150 nm) for NanoSIMS and ultrathin (70 nm) for TEM analysis. NanoSIMS sections were placed onto antimony-doped silicon wafer platelets (7,1 x 7,1 x 0,75 mm; Active Business Company GmbH, Brunnthal, Germany). TEM sections were placed on Formvar®-coated slot grids and stained with 0.5% uranyl acetate and 3% lead citrate prior to imaging with a Zeiss® Libra 120 transmission electron microscope. NanoSIMS and TEM images were overlaid using the GIMP® software.

**NanoSIMS measurements, data evaluation and statistical analysis**

All measurements were carried out on a NS 50L instrument (Cameca, Gennevilliers, France). Data were acquired as multilayer image stacks obtained by sequential scanning of a finely focused Cs^+^ ion beam (approx. 80 nm probe size) and simultaneous detection of secondary ions as well as secondary electrons. The detectors of the multicollection assembly were positioned to enable parallel detection of ^12^C^−^, ^13^C^−^, ^12^C_2_^−^, ^12^C^13^C^−^, ^12^C^14^N^−^, ^31^P^−^ and ^32^S^-^ secondary ions. The electrostatic lenses and deflectors inside the spectrometer were adjusted to achieve a mass resolving power (MRP) of > 10,000 (according to Cameca’s definition) for detection of C_2_^–^ secondary ions. During acquisition, secondary ion beam drift was corrected by automatic beam centering (utilizing the ^12^C_2_^−^ signal as reference) as well as automatic peak centering for each of the recorded secondary ion species (utilizing the ^12^C^-^, ^12^C_2_^-^ and ^12^C^14^N^-^ signals as reference signals for the ^13^C^-^, ^12^C^13^C^-^ and ^31^P^-^, ^32^S^-^ signals, respectively). Scanning areas were in the range from 40 × 40 to 60 × 60 µm^2^ at typically 512 × 512 pixel image resolution and 7.5 to 10 msec dwell time per pixel and cycle. The total per pixel dwell time ranged from 100 to 150 msec per image stack.

Image data were evaluated using the WinImage software package (version 2.0.8) provided by Cameca. Prior to stack accumulation, the individual images were aligned to compensate for positional variations arising from primary ion beam and/or sample stage drift. Secondary ion signal intensities were dead-time corrected on a per-pixel basis and corrected for quasi simultaneous arrival (QSA) of C^-^ and C_2_^-^ secondary ions at the detectors on a per-ROI basis. The QSA correction was performed according to the formalism suggested by Slodzian et al. (2004), applying sensitivity factors of 1.1 and 1.06 for C^-^ and C_2_^-^ ions, respectively (experimentally determined by measurements on dried yeast cells, data not shown). ^13^C/(^12^C + ^13^C) isotope fractions were calculated from ^12^C_2_^-^ and ^12^C^13^C^-^ signal intensities via ^13^C/(^12^C + ^13^C) = ^12^C^13^C^-^/(2*^12^C_2_^-^ + ^12^C^13^C^-^) and cross-checked by the values obtained from the ^12^C^-^ and ^13^C^-^ signal intensities via ^13^C/(^12^C + ^13^C) = ^13^C^-^/(^12^C^-^ + ^13^C^-^). Due to superior counting statistics achieved with the molecular secondary ion species, all ROI data and isotope fraction distribution images displayed in the manuscript were inferred from the ^12^C_2_^-^ and ^12^C^13^C^-^ signal intensities. To visualize and evaluate the relative sulfur content, ^32^S^-^ signal intensities were normalized to the corresponding C^-^ signal intensities (i.e. sum of the ^12^C^-^ and ^13^C^-^ signal intensities). C^-^ was utilized for normalization due to the high abundance of carbon in the matrix and the similar energy and angular distribution of atomic secondary ion species, which enables largely eliminating (instrument-related) acquisition-to-acquisition variability of the S^-^ signal intensity.

Individual regions of interest (ROI) were manually defined based on the morphological features identifiable in the ^12^C^14^N^−^, ^31^P^−^ and/or ^32^S^−^ signal intensity distribution maps as well as the structural information gained from the TEM images. 50 to 150 individual symbionts were analyzed per treatment, each symbiont defining one ROI. The host ROI (N=50 per treatment) were defined manually in the host cytoplasm.

Since most data departed from normal distribution (according to Shapiro-Wilk tests) Wilcoxon-Mann-Whitney significance testing with exact permutations was conducted to identify significant differences in the medians of two individual data sets (Bergmann *et al.,* 2000). The Scheffe method was applied to compare multiple data sets (Scheffe, 1959). All statistical calculations were performed utilizing *Agricolae* (V1.2-3) and *coin* (V1.1-1) software packages, implemented in R (Ihaka & Gentleman 1996, V3.2.2) and accessed via the Comprehensive R Archive Network (CRAN). Data are presented as median and interquartile range (IQR), which were evaluated by applying the "Tukey's hinges" quartile method (Tukey 1977).

**Acid phosphatase ultracytochemistry**

Freshly collected colonies (n=3) were briefly fixed in 2% glutaraldehyde and 1% paraformaldehyde in sodium cacodylate buffer (0.1 mol L^-1^; 1100 mOsm L^-1^; pH = 7.2) for 1 h and washed in the same buffer several times. Note that the fixation which is necessary to preserve the sample ultrastructure might diminish the enzymatic activity. The fixed colonies were then immersed in a 3.3 mg mL^-1^ sodium ß-glycrophosphate solution saturated with 3.3 mmol L^-1^ lead nitrate at 37°C for 25 min, which leads to the formation of electron-dense lead precipitates through acid phosphatase activity (Gomori, 1952). The specificity of the reaction was tested on three colonies by omitting sodium ß-glycrophosphate from the medium. All specimens were subsequently rinsed with an acetate buffer solution (sodium acetate 50 mmol L^-1^, acetic acid 15 mmol L^-1^; pH = 5.5) followed by 3.5% acetic acid before ethanol series dehydration and embedding in medium grade LR White resin. Ultrathin sections (70 nm) were imaged - without further staining - with a Zeiss® Libra 120 transmission electron microscope.

**16S rRNA gene sequencing and fluorescence *in situ* hybridization (FISH)**

In order to confirm the identity of the symbiont, 16S rRNA gene clone libraries were obtained for three colonies, with the lower part cut off to avoid contamination with other microbes and rinsed in 0.2 µm filtered seawater several times prior freezing in liquid nitrogen. Template DNA for PCR was obtained by homogenizing each colony (N=5) with a vortex mixer in Tris-HCl buffer (10 mmol L^-1^; pH 8.5) for 5 s. General primers 27F (Lane 1991) and 1492R (Loy *et al.,* 2005), which target most bacteria, were used. The reaction mixture (1 µL sample in a total volume of 50 µL) contained dNTPs 0.2 mmol L^-1^, Taq polymerase 0.02 U/µL, primers 0.5 µmol L^-1^, MgCl_2_ 2 mmol L^-1^, and BSA 0.01 mg/mL. The PCR was run on an Eppendorf MasterCycler temperature gradient thermocycler with an annealing temperature of 55°C. Amplified products were ligated and cloned with the TOPO TA Cloning Vector pCR 2.1 and TOP10 chemically competent cells (Invitrogen) according to the manufacturer’s instructions. After overnight growth, plates were kept at 4°C for 2 h and up to ten clones with the right insert length of approximately 1500 bp (screened using vector-specific primers M13F and M13R) from each initial sample were capillary sequenced (Microsynth). The chromatograms were manually proofread with FinchTV 1.5 (Geospiza) and a consensus sequence between forward and reverse was obtained with Codon Code Aligner 3.7.

For FISH, four colonies were fixed in absolute ethanol immediately after collection in the natural environment prior to embedding in the medium-grade LR White resin. Polymerization was performed under nitrogen atmosphere at only 40°C for three days. Semithin sections (0.5 µm) were mount on gelatin-coated slides. The hybridization solution (0.9 mol L^-1^ NaCl, 20 mmol L^-1^ Tris/HCl pH 8.2, 0.01% SDS, 10% formamide) was applied onto the section in a 30 µL drop containing 1 ng mL^-1^ of each oligonucleotide probe. Hybridization was performed in a humid chamber at 46°C for 3 h. Washing was performed under stringent conditions at 48°C for 15 min (Manz *et al*., 1992). A mix of EUB338-I, EUB338-II, EUB338-III (Daims *et al*., 1999) and Arch915 (Stahl and Amann, 1991) – all labeled with Cy5 – was applied to target most bacteria and archaea. A previously published symbiont-specific probe (ZNS196; Rinke *et al.,* 2006) was modified to account for a single mismatch found in the probe target site of all five newly retrieved symbiont 16S rRNA gene sequences, and used as Cy3-labeled derivative to target the ectosymbiont *Cand.* Thiobios zoothamnicoli (ZNS196_mod, Supplementary Figure 3). For this probe, 10% formamide concentration was determined as optimal after performing a formamide concentration series experiment from 0 to 30%. Nonsense probes labeled in Cy3 and Cy5 were also applied to all slides to control for false positive signals due to autofluorescence or unspecific probe binding, but no signal were observed in these controls. Multiple micrographs with overlapping fields were taken to cover each entire colony with a 100x objective on an epifluorescence microscope (Axio Imager M2, Carl Zeiss) equipped with a black-and-white high-resolution camera (AxioCam MRm, Carl Zeiss). The micrographs (294 in total) were then assembled with the image stitching program MICE® to produce three composite pictures of each entire colony with the DAPI, Cy3 and Cy5 signals, respectively. The three composite pictures were then uploaded to the GIMP® software where they were overlaid. For each digestive vacuole present on the section, we counted the symbiont-specific FISH signals and compared numbers to those labeled only with a EUB_mix_. and Archaea probe mix to estimate the composition of the host diet.

**References**

Bergmann R, Ludbrook J, Spooren WPJM. (2000). Different outcomes of the Wilcoxon-Mann-Whitney test from different statistics packages. *Am Stat* **54**:72-77.

Daims H, Brühl A, Amann R, Schleifer K-H, Wagner M. (1999). The domain-specific probe EUB338 is insufficient for the detection of all bacteria: development and evaluation of a more comprehensive probe set. *Syst Appl Microbiol* **22:**434–444.

Erikson A and Fisher SK. (1990). Tritiated uridine labeling of the retina: Variations among retinal quadrants, and between right and left eyes. *Exp Eye Res* **51**:145-152.

Goldammer H, Hollergschwandtner E, Elisabeth NH, Frade PR, Reipert S (2016) Automatized freeze substitution of algae accelerated by a novel agitation module. *Protist* **167**:369-376.

Gomori G. (1952). Microscopic Histochemistry: Principles and Practice. University of Chicago Press: Chicago.

Ihaka R, Gentleman R. (1996). R: A language for data analysis and graphics. *J Comput Graph Stat* **5**:299-314.

Lane DJ. (1991). "16S/23S rRNA sequencing". In: Stackebrandt E and Goodfellow M. (Eds). Nucleic acid techniques in bacterial systematics. John Wiley & Sons Ltd: Chichester, pp 115-175.

Loy A, Schulz C, Lücker S, Schöpfer-Wendels A, Stoecker K, Baranyi C*, et al.* (2005). 16S rRNA gene-based oligonucleotide microarray for environmental monitoring of the betaproteobacterial order "Rhodocyclales". *Appl Environ Microbiol* **71**:1373-1386.

Manz W, Amann R, Ludwig W, Wagner M, Schleifer K-H. (1992). Phylogenetic oligonucleotide probes for the major subclasses of proteobacteria: problems and solutions. *Syst Appl Microbiol* **15:**593–600.

McDowell EM, Trump BF. (1976). Histologic fixatives suitable for diagnostic light and electron microscopy. *Arch Pathol Lab Med*. **100**:405-414.

Rinke C, Schmitz-Esser S, Stoecker K, Nussbaumer AD, Molnár DA, Vanura K*, et al.* (2006). "*Candidatus* thiobios zoothamnicoli," an ectosymbiotic bacterium covering the giant marine ciliate *Zoothamnium niveum*. *Appl Environ Microbiol* **72**:2014-2021.

Roger AW. (1979). Techniques of autoradiography 3^rd^ Edn. Elsvier North-Holland Biomedical Press: Amsterdam.

Scheffe H. (1959). The Analysis of Variance. John Wiley & Sons: New York.

Slodzain G, Hillion F, Stadermann FJ, Zinner E. (2004). QSA influences on isotopic ratio measurements. *Appl. Surf. Sci*. **231-232:** 874-877

Stahl DA and Amann R. (1991). Development and application of nucleic acid probes in bacterial systematics, p. 205-248. In E. Stackebrandt and M. Goodfellow (ed.), Nucleic acid techniques in bacterial systematics. Wiley & Sons Ltd., Chichester, England.

Tukey JW. (1977). Exploratory Data Analysis. Addison-Wesley: Boston

Supplementary Figures and Tables:


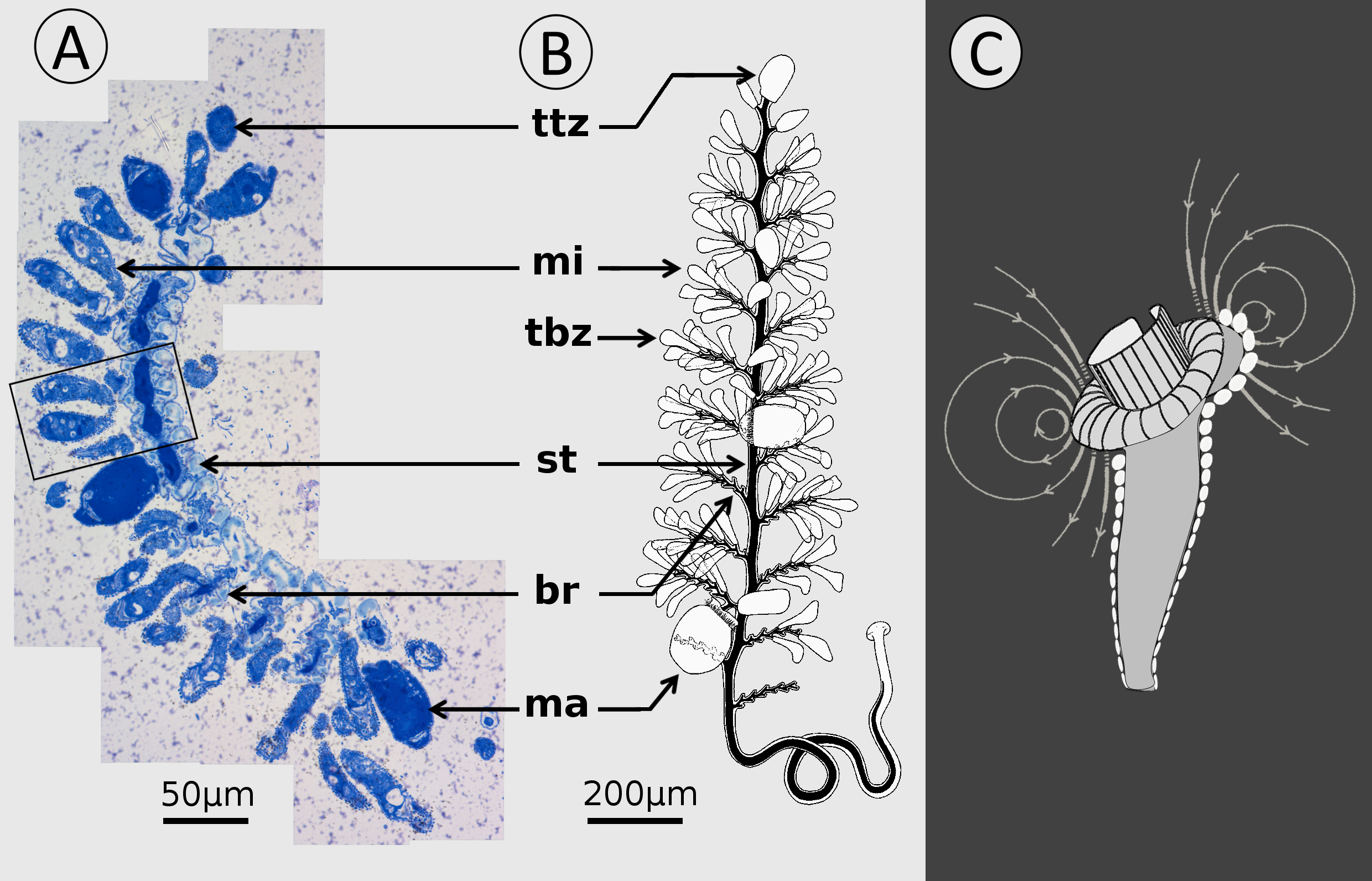


**Supplementary Figure 1: Anatomy of the *Zoothamnium niveum* colony. A**. Composite picture of a colony section from the autoradiography experiment. The detail of the two microzooids (mi) in the rectangle is given on Supplementary Figure 2. Here the terminal top zooid (ttz) and two macrozooids (ma) are visible. The section plan also passes through the stalk (st) and some branches (br). **B**. Drawing of a colony from the redescription of *Z. niveum* (Bauer-Nebelsick *et al*., 1996a). tbz: top branch zooid. **C**. Schematic drawing of a single microzooid modified from Bauer-Nebelsick *et al*. (1996a). The oral ciliature creates a water current allowing the cell to filter feed. The two symbiont cell morphotypes are represented here on the surface of the microzooid. Coccoid-shaped symbiont cells are on the oral part, and rod-shaped on the aboral part of the microzooid.


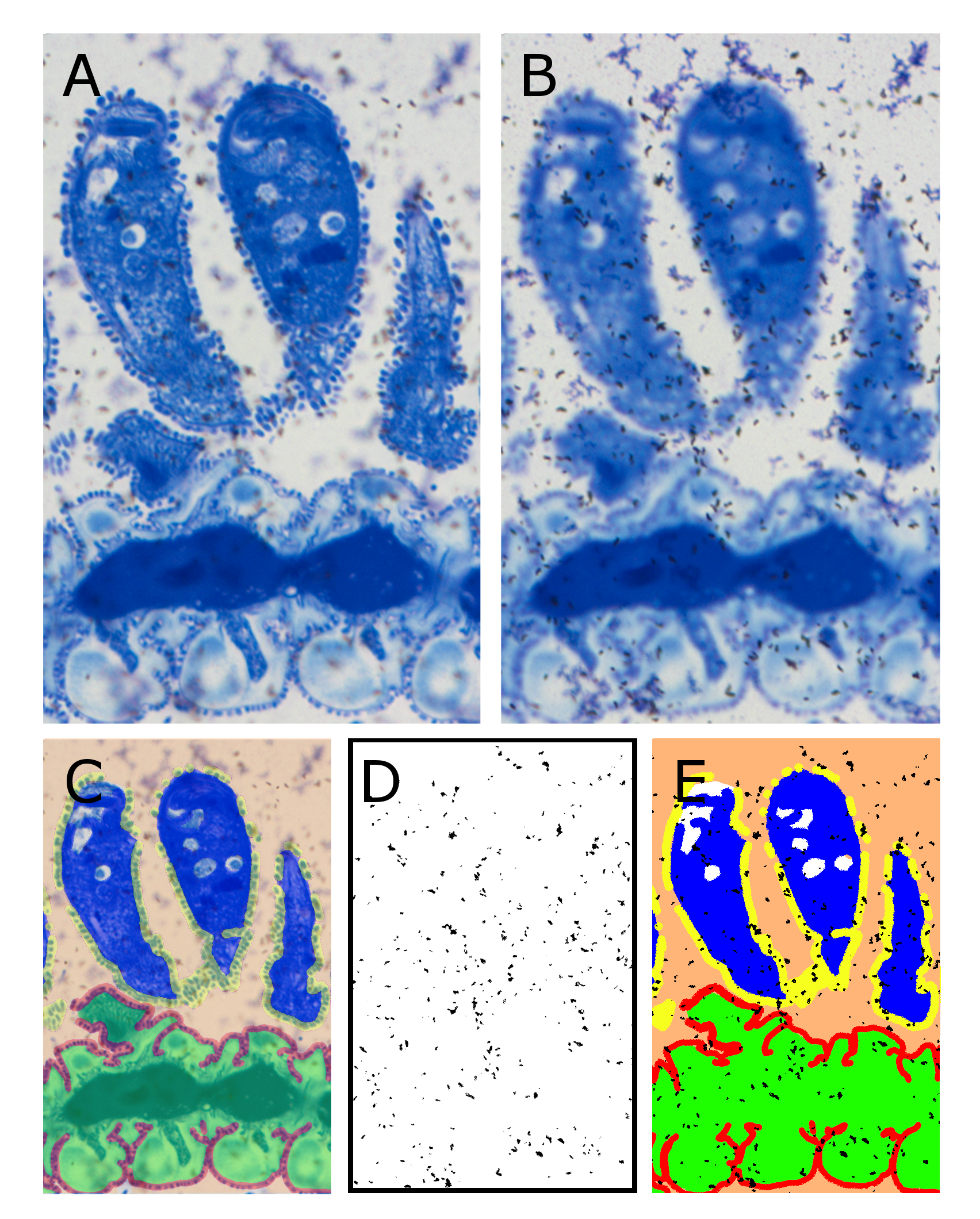


**Supplementary Figure 2: Detail of two microzooids and stalk and the different areas analyzed on the autoradiographs. A**. Light micrograph taken with the focus on the colony. **B**. The same field of view with the focus on the autoradiographic emulsion showing the silver grains formed by incorporated ^14^C. **C**. The different areas are painted in colors on the image with the colony focus. Symbiont cells attached to microzooids are painted yellow, symbiont cells attached to stalk red, ciliate microzooid cytoplasm blue (digestive vacuoles and cilia are omitted from the analysis). The stalk is painted green. The empty resin area directly around the cells is dark salmon and considered background. **D**. The silver grains from image B are extracted using the “selection by color” tool in GIMP® and a black –and-white image is created. **E**. Overlay of the different defined areas and the silver grains. For each area the actual grain density (AGD) is analyzed as the number of black pixels expressed as a percentage of total pixels in the area.

**
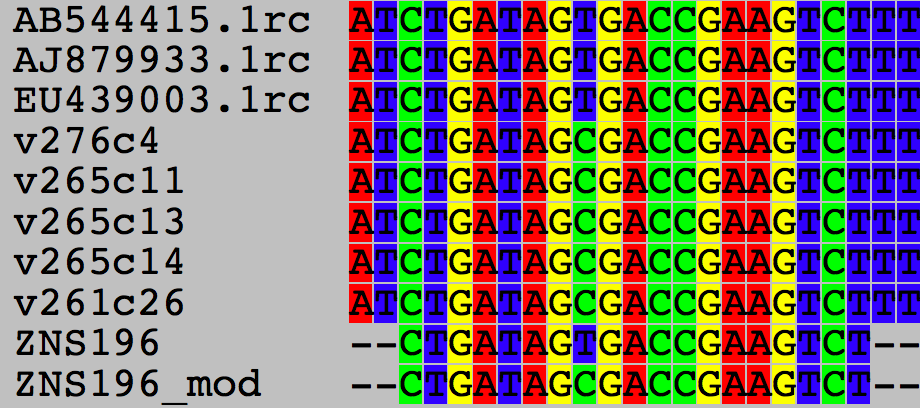
**

**Supplementary Figure 3:** Alignment of the binding sites of probes ZNS196 (Rinke *et al.*, 2006, 2009; Kawato et al. 2010) and ZNS196_mod (this study) within 16S rRNA sequences affiliated with Candidatus Thiobios zoothamnicoli (AB544415 from Bay of Tokyo, Japan (Kawato et al. 2010); AJ879933 from Twin Cays, Belize (Rinke et al. 2006); EU439003 from Calvi, France (Rinke et al. 2009); v276c4, v265c11, v265c13, v265c14, v261c26 from Portorož, Slovenia, this study). Probe sequences are shown reverse and complementary.


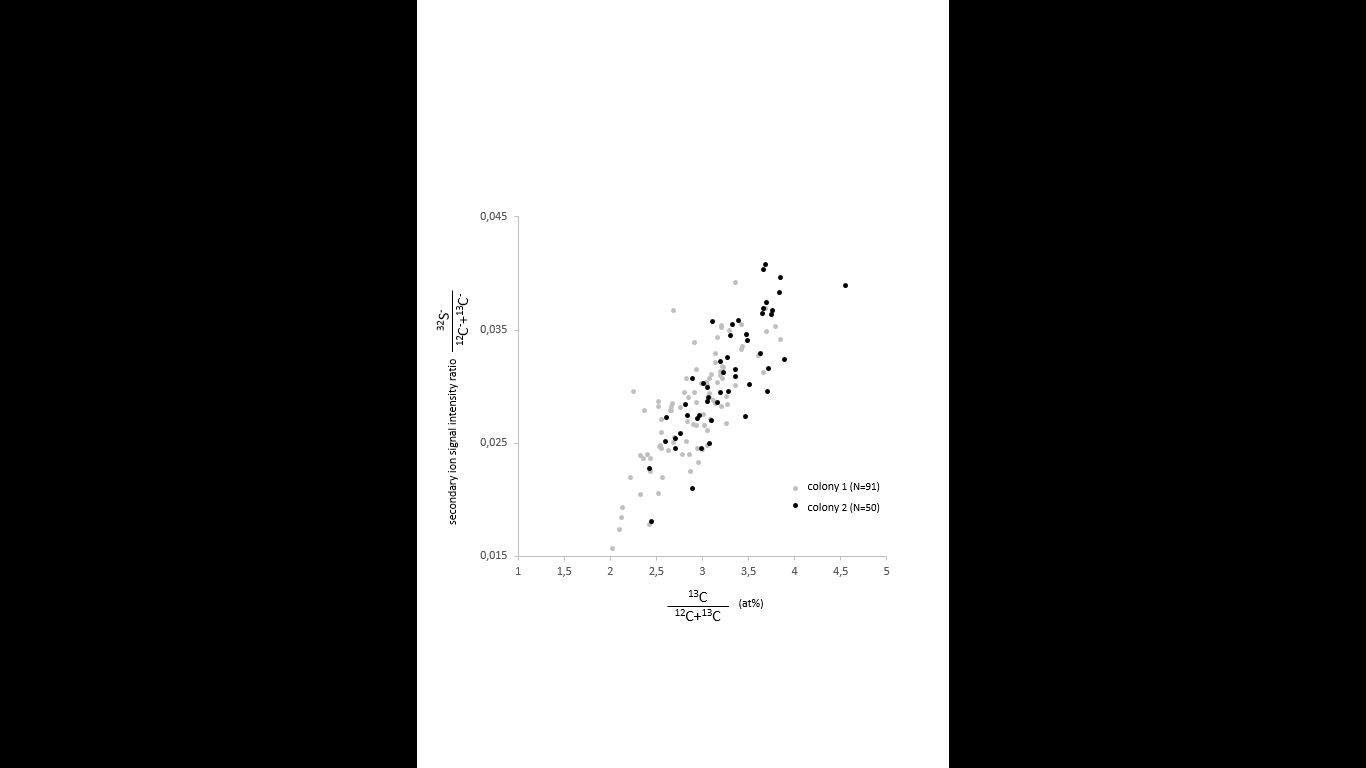


**Supplementary Figure 4: Relative sulfur *vs.* ^13^C label content as inferred from NanoSIMS analysis of microzooid symbionts in the two colonies of the sulfidic pulse in the presence of ^13^C bicarbonate.** Scatter plot of the ROI specific C^-^ normalized ^32^S^-^ secondary ion signal intensity *vs.* the ^13^C/(^12^C+^13^C) isotope fraction (given in at%) for each of the 141 symbiont cells. The correlation between the sulfur content and ^13^C incorporation is positive.

**Supplementary Table 1: Summary of incubations**.

| **incubation** | **pre-treatment** | **pulse time** | **tracer added in the pulse** | **[ΣH_2_S]**  **during the pulse (µmol L^-1^)** | **chase time** | **[ΣH_2_S]**  **during the chase (µmol L^-1^)** | **temperature**  **(°C)** |
| --- | --- | --- | --- | --- | --- | --- | --- |
| ^14^C sulfidic pulse | no | 25 min | NaH^14^CO_3_ | 12.2 | - | - | 22.2 |
| ^14^C oxic pulse | no | 25 min | NaH^14^CO_3_ | 0 | - | - | 22.2 |
| 24h oxic + ^14^C oxic pulse | 24h in oxic seawater | 25 min | NaH^14^CO_3_ | 0 | - | - | 22.2 |
| ^14^C sulfidic pulse - chase | no | 25 min | NaH^14^CO_3_ | 12.2 | 6h | 12.4 | 22.2 |
| ^14^C dead control | killed in ethanol | 25 min | NaH^14^CO_3_ | 12.2 | - | - | 22.2 |
| ^14^C natural carbon control | no | 25 min | - | 12.2 | - | - | 22.2 |
| ^13^C sulfidic pulse | no | 3 h | NaH^13^CO_3_ | 27.1 | - | - | 24.5 |
| 24h oxic + ^13^C oxic pulse | 24h in oxic seawater | 3h | NaH^13^CO_3_ | 0 | - | - | 24.5 |
| ^13^C dead control | killed in ethanol | 3h | NaH^13^CO_3_ | 27.1 | - | - | 24.5 |
| ^13^C natural carbon control | no | 3h | - | 27.1 | - | - | 24.5 |

**Supplementary Table 2: Analysis of the digestive vacuole content in the four replicate colonies.** All prokaryotes observed in digestive vacuoles were counted. Here the number of microbial cells labeled with the EUB338_mix_/Arch915 probes (total number of microbes) and the number of bacteria simultaneously labeled with the symbiont specific ZNS196_mod probe are presented.

| **colony #** | **EUB338_mix_/Arch915** |  | **ZNS196_mod** | **% symbiont** |
| --- | --- | --- | --- | --- |
| **1** | 36 |  | 35 | 97.2 |
| **2** | 12 |  | 10 | 83.3 |
| **3** | 26 |  | 22 | 84.6 |
| **4** | 14 |  | 13 | 92.9 |
